# Early evidence of extra‐masticatory dental wear in a Neolithic community at Bestansur, Iraqi Kurdistan

**DOI:** 10.1002/oa.3162

**Published:** 2022-09-27

**Authors:** Sam Walsh

**Affiliations:** ^1^ School of Natural Sciences University of Central Lancashire Preston UK

**Keywords:** eastern Fertile Crescent, extra‐masticatory dental wear, Neolithic, teeth as tools

## Abstract

This paper presents the first evidence of extra‐masticatory dental wear from Neolithic Bestansur, Iraqi Kurdistan (7700–7200 BC). Bestansur is a rare, recently excavated burial site of this period in the Zagros region, of Iraqi Kurdistan. A total of 585 teeth from 38 individuals were analyzed for features indicative of activities including oblique wear planes, notches, grooves, and chipping. Indications of extra‐masticatory wear were found in 27 of 38 individuals, and 277 of 585 teeth (47%) available for study. The most frequent features were chipping and notches suggesting activities such as processing fibers by using the teeth as a “third hand.” Evidence for these wear features was present in both males, females, and in children aged five and older. These aspects of childhood life‐course and dentition are rarely investigated. The presence of dental wear features in the deciduous dentition can indicate an age range at which activities began in different groups and highlights the importance of including juvenile remains in such studies. The variety of forms of dental wear may relate to the mixed diet and activities of these people. This study adds to our understanding of human behaviors and socio‐cultural aspects of life during this transitional period.

## 1 INTRODUCTION

Dental wear is often categorized as masticatory wear, caused by diet and sustenance, or extra‐masticatory wear, where teeth are used as tools or for other activities. Dental wear resulting from activities produces alterations to the teeth, which include features such as chipping, notching, polished areas of enamel such as lingual surface attrition of maxillary anterior teeth (LSAMAT), and occlusal and interproximal grooves (Bonfiglioli et al., 2004; Lukacs & Pastor, 1988; Ricci et al., 2016; Turner & Machado, 1983). In addition, extremely oblique, or differential wear may also indicate extra‐masticatory wear (Alt & Pichler, 1998; Molnar, 2008; Scott & Jolie, 2008).

Alterations to teeth may be incidental or intentional, differentiating between these alterations can be difficult, as both categories can affect teeth in ways that overlap (Lozano et al., 2013; Stojanowski et al., 2016). Incidental alterations may be caused by food preparation, trauma, hygiene practices, and activities such as processing fibers (Alt & Pichler, 1998; Bonfiglioli et al., 2004). Intentional modifications relate to socio‐cultural or therapeutic alterations (Verdugo et al., 2020) including purposeful tooth removal, both therapeutic and nontherapeutic (De Groote & Humphrey, 2017; Pardoe & Durband, 2017; Palefsky, 2019; Willman, 2016) or other interventions such as drilling or filing (Arcini, 2005; Coppa et al., 2006; Oxilia et al., 2017).

Dental wear from mastication is affected by food composition, preparation, and inclusions. Masticatory wear usually leads to the formation of wear facets and microwear comparable across the dentition (Alt & Pichler, 1998; Kaidonis, 2008; Schmidt et al., 2019). In contrast, wear patterns from using teeth as tools are irregular in severity and distribution (Lukacs & Pastor, 1988).

Evidence for dental wear features has been found worldwide with examples seen in hominids (Ungar et al., 2001), prehistoric groups (Fidalgo et al., 2020; Sperduti et al., 2018), anthropological studies (Berbesque et al., 2012; Garve et al., 2017), and in Neolithic groups in Poland (Lorkiewicz, 2011), Pakistan (Lukacs & Pastor, 1988), Sweden (Molnar, 2008), and of most relevance to this study at Abu Huyreyra, Syria (Molleson & Jones, 1991). There has been little study of Neolithic dentition in the Zagros region, and few studies have investigated all aspects of extra‐masticatory wear within Southwest Asia.

Juvenile individuals are rarely included in studies of extra‐masticatory wear, due to the complicating factors of deciduous dentition which include mixed dentition, variation in eruption sequences per population, weaning foods, and timing of weaning (Beck & Smith, 2019; Estalrrich & Marín‐Arroyo, 2021).

### 1.1 Aims and objectives

Through the analysis of the Bestansur dentition, this study aims to investigate the frequency and co‐occurrence of extra‐masticatory dental wear features, social bias in affected individuals, and the potential causes of these features. The objectives are to examine the dentition through macroscopic and microscopic analyses at both the individual and assemblage scale to demonstrate the presence or absence of extra‐masticatory dental wear features.

### 1.2 Archeological background

**FIGURE 1:**
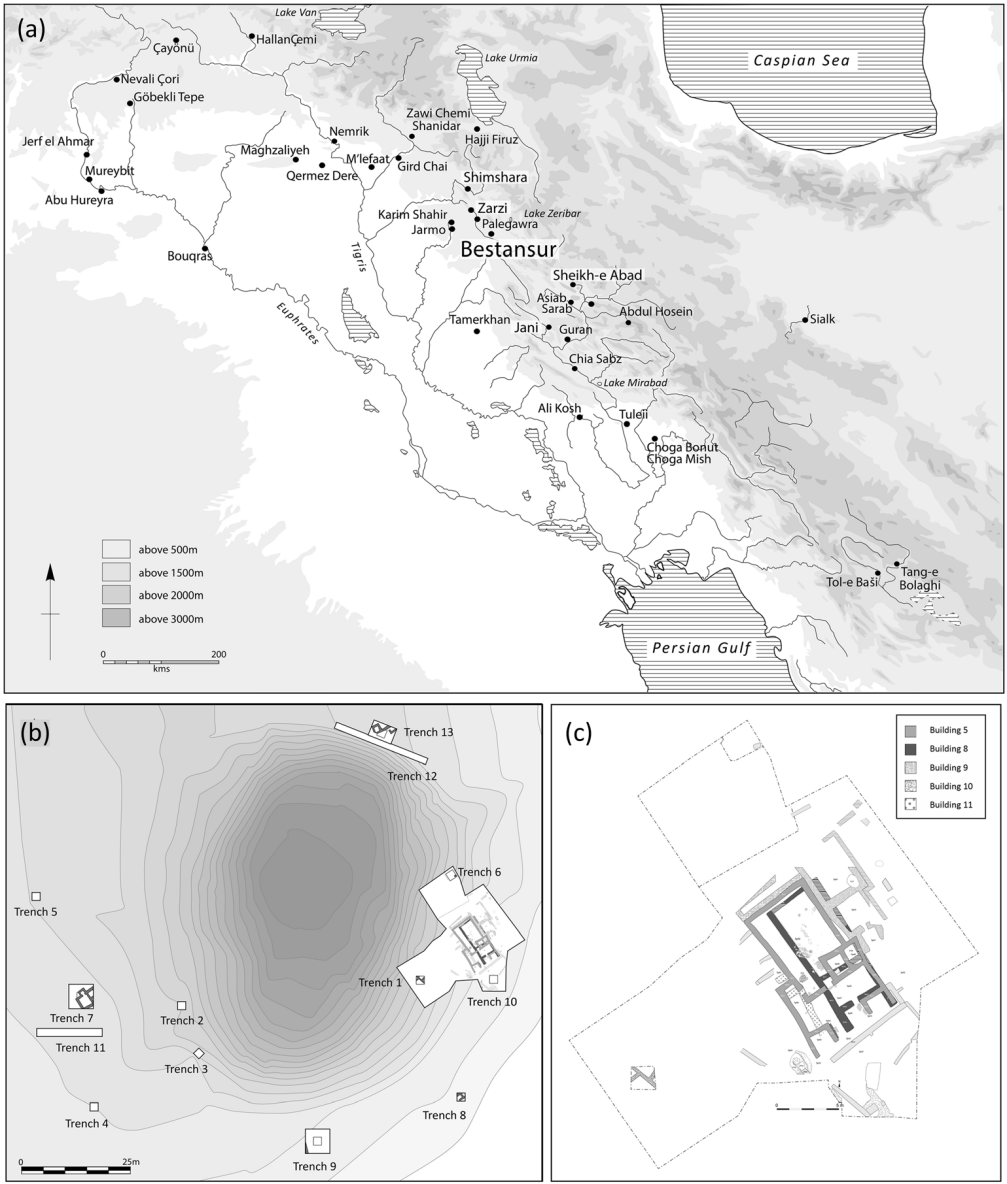
(a) Location map of Bestansur, (b) contour plan of Bestansur mound showing location of trenches, (c) plan of trench 10

The site of Bestansur is on the Shahrizor plain, 33 km southeast of Sulaimaniya, Iraqi Kurdistan (Figure 1). Excavations of Neolithic levels have revealed mudbrick structures, fire installations, burials, and areas of activities such as lithic working, and the site dates from c. 7700 to 7100 cal BC (Flohr et al., 2020; Matthews et al., 2019). Numerous burial deposits across the site have revealed human remains from at least 99 individuals, predominantly from two large buildings. The burial deposits range from complete articulated individuals to partially articulated comingled successive interments (Walsh, 2020).

The zooarcheological evidence from Bestansur indicates a community in the early phases of adopting goat and pig management, while utilizing a wide range of wild animals from wetlands, woodlands, and open areas (Bendrey et al., 2020; de Groene et al., 2021). Fecal evidence of grazing of herbivores and wild boar or pig provide further evidence of early animal management (Elliot, 2020). Small finds demonstrate evidence for the acquisition of objects and materials from both local and more distant sources. Ground stone tool types from Bestansur were likely used for food processing, hunting, and fishing (Matthews et al., 2019). In summary, the people of Bestansur were a transitional Neolithic group harnessing a variety of resources both for dietary and material purposes as part of the adoption of sedentism.

## 2 MATERIALS AND METHODS

### 2.1 Materials

A total of 819 teeth were available for study; 234 teeth were excluded due to lack of preservation or eruption, leaving 585 permanent and deciduous teeth for analysis. A total of 475 teeth were associated with distinct individuals, a further 110 teeth were disarticulated but included in the sample due to their potential to increase understanding of tooth wear in this group.

A total of 99 Neolithic individuals have been excavated so far, but due to the absence of dentition through development or post‐mortem tooth loss it was only possible to analyze the dentition of 38 individuals. There were 18 adults and 20 juveniles, ranging in age from around 4 to 50+ years. Determination of sex was possible in 16 adults and four adolescents resulting in equal numbers of males and females (see Table S1 and Walsh, 2020).

### 2.2 Methods

All teeth were assessed macroscopically for dental wear; specimens with features were selected for further analysis using light microscopy. A subset of these specimens underwent scanning electron microscopy (SEM) (FEI Quanta FEG 600) for clarification. These teeth were analyzed directly due to their fragile state, in low vacuum mode with a magnification of 54× and a working distance of 11.2 to 12.2 mm.

The teeth were examined for features including notches, occlusal grooves, chipping, angled wear, and interproximal grooves using the methods associated with descriptions below.

A notch is defined as an indent at the incisal or occlusal edge, which may extend over the whole occlusal surface; notches have greater breadth than depth and are oriented on the median line (Bonfiglioli et al., 2004). Occlusal grooves appear as a narrow linear groove oriented transversely across the occlusal surface (Molnar, 2011). Interproximal grooves are located on the interproximal surfaces, near the cemento‐enamel junction (Frayer, 1991; Lozano et al., 2013; Lukacs & Pastor, 1988). Chipping is defined as an irregular microfracture of enamel or dentine at the tooth margins (Bonfiglioli et al., 2004; Lozano et al., 2017). These fractures occur when the margins are exposed to high bite forces on hard objects (Scott & Winn, 2011; Willman, 2016, p. 50). These attributes are recorded by presence or absence only, as severity is likely to vary between populations.

In addition, the teeth were examined for oblique and differential wear using Molnar's (1971) categories, to allow categorization of directionality and extremes of wear. Skinner (1997) was used to assess the levels of dental wear in deciduous teeth and gave similar results to the lower wear stages of Molnar (1971).

Taphonomic processes that could affect interpretations of dental wear, including fracture types (Scott & Winn, 2011), heat alteration (Schmidt, 2015), staining, and surface cracking and delamination (Dirks et al., 2015; Hughes & White, 2009), were ruled out through microscopic analysis.

### 2.3 Limitations

The skeletal remains from Bestansur are often poorly preserved due to soil type, resulting in fragmented maxillary and mandibular bones. It was not possible to rule out ante‐mortem movement of teeth such as lingual tilting as a cause of wear (Reinhardt, 1983). Due to these issues, angles of wear were not measured quantitatively (Smith, 1984). There is a lack of taphonomic methodology for teeth (Willman et al., 2019), and methods for the analysis of deciduous tooth wear are also limited (Beck & Smith, 2019; Hernando et al., 2020).

## 3 RESULTS

Of the total 585 teeth, 277 (47.3%) were affected by some aspect of extra‐masticatory wear (Table 1). The types of extra‐masticatory wear and features seen in the Bestansur assemblage include oblique wear, notching, occlusal grooves, and chipping. Of the 38 individuals analyzed, 27 have dental wear features from extra‐masticatory activities, of these 10 are juveniles (see Table 2). Aside from oblique wear, the most frequent features were chipping, with 136 teeth and 18 individuals affected. Notched teeth were the next most frequent feature with 41 teeth and seven individuals affected. Occlusal grooves were less common; this may be due to increasing masticatory wear through the life‐course.

**TABLE 1:** Number of individuals and teeth affected by extra‐masticatory wear

Category	Total included	Total affected	% Affected
Total teeth	585	277	47.35
Permanent teeth	453	200	44.15
Deciduous teeth	132	77	43.18
Individuals	38	27	71.05

**TABLE 2:** Number (*N*) and percentage of oblique wear and features caused by extra masticatory wear affecting teeth and individuals

		Oblique wear		Notches		Occlusal/transverse grooves		Chipping	
	Total analysed	*N*	%	*N*	%	*N*	%	*N*	%
Permanent teeth	453	115	25.3	33	7.2	12	2.6	115	25.3
Deciduous teeth	132	21	15.9	8	6	0	0	21	15.9
Adult individuals	18	13	72.2	4	22.2	3	16.6	9	50
Juvenile individuals	20	7	35	3	15	0	0	9	45

### 3.1 Forms of wear

**FIGURE 2:**
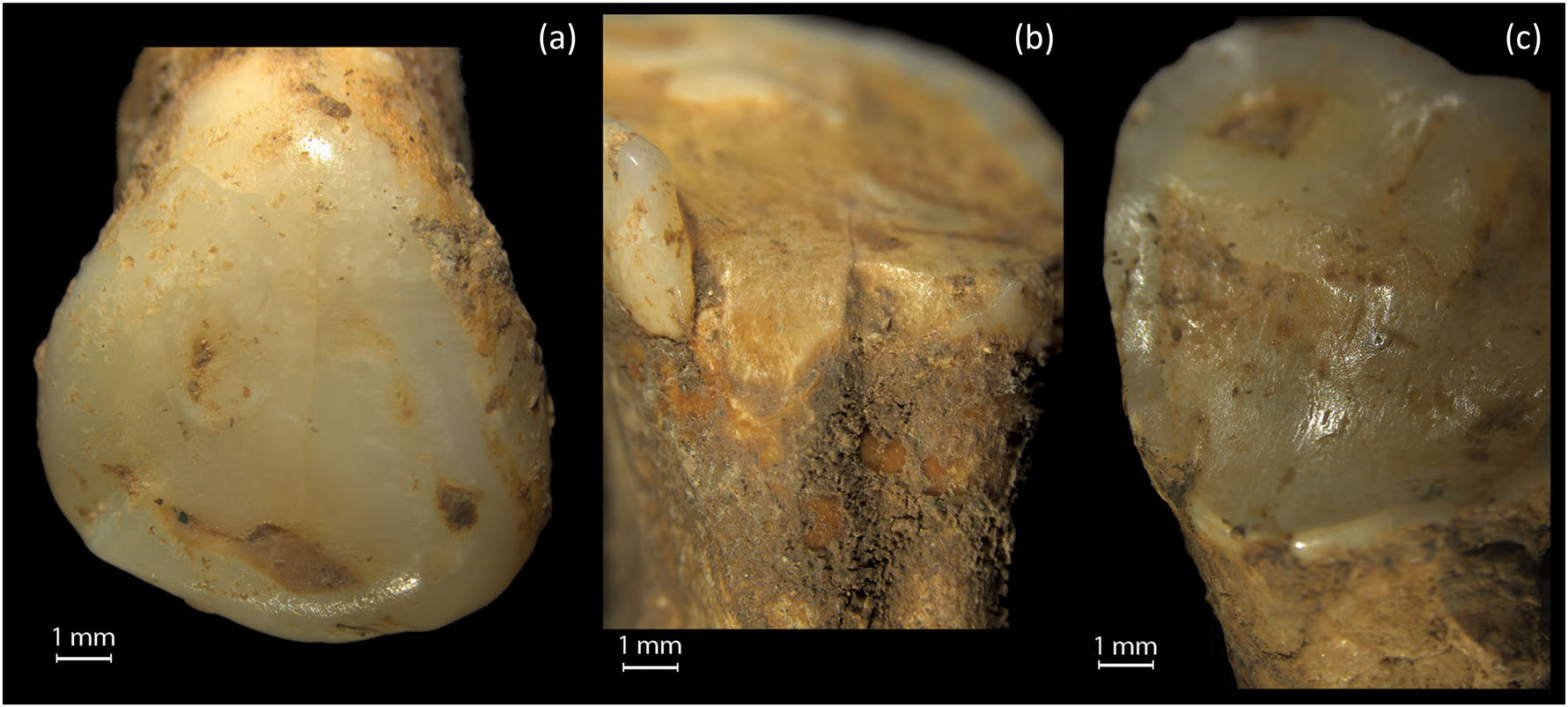
Examples of oblique dental wear, (a) tooth 300 (SK1788) upper left canine lingual aspect; (b) tooth 297 (SK1788) upper right M1 lingual aspect; (c) tooth 844 (SK1868‐4) upper left M2 lingual aspect [Colour figure can be viewed at wileyonlinelibrary.com]

Oblique dental wear was analysed to assess differential wear, and the impact of diet or activities. Oblique wear planes affected 165 (36.4%) permanent teeth and 31 (26.9%) deciduous teeth. Oblique wear was common in adult individuals (72%) with males and females affected equally (Figure 2).

Buccal‐lingual wear (from the outside of the mouth directed inwards) was the most common plane of wear affecting 80 teeth or 17.6% of the worn permanent teeth (see Table S2). Small amounts of buccal‐lingual and distal‐mesial angled wear affected four juvenile individuals aged 4 to 9 with mixed dentition that have additional features of extra‐masticatory wear.

In terms of differential wear, there were two adult individuals of interest. A young adult male (SK1625‐1) had high levels of anterior wear and fracturing of the incisors, in addition to differential wear to the right side. A young adult female (SK1788) had increased levels of wear to the upper first molars, which do not match the lower molars.

**FIGURE 3:**
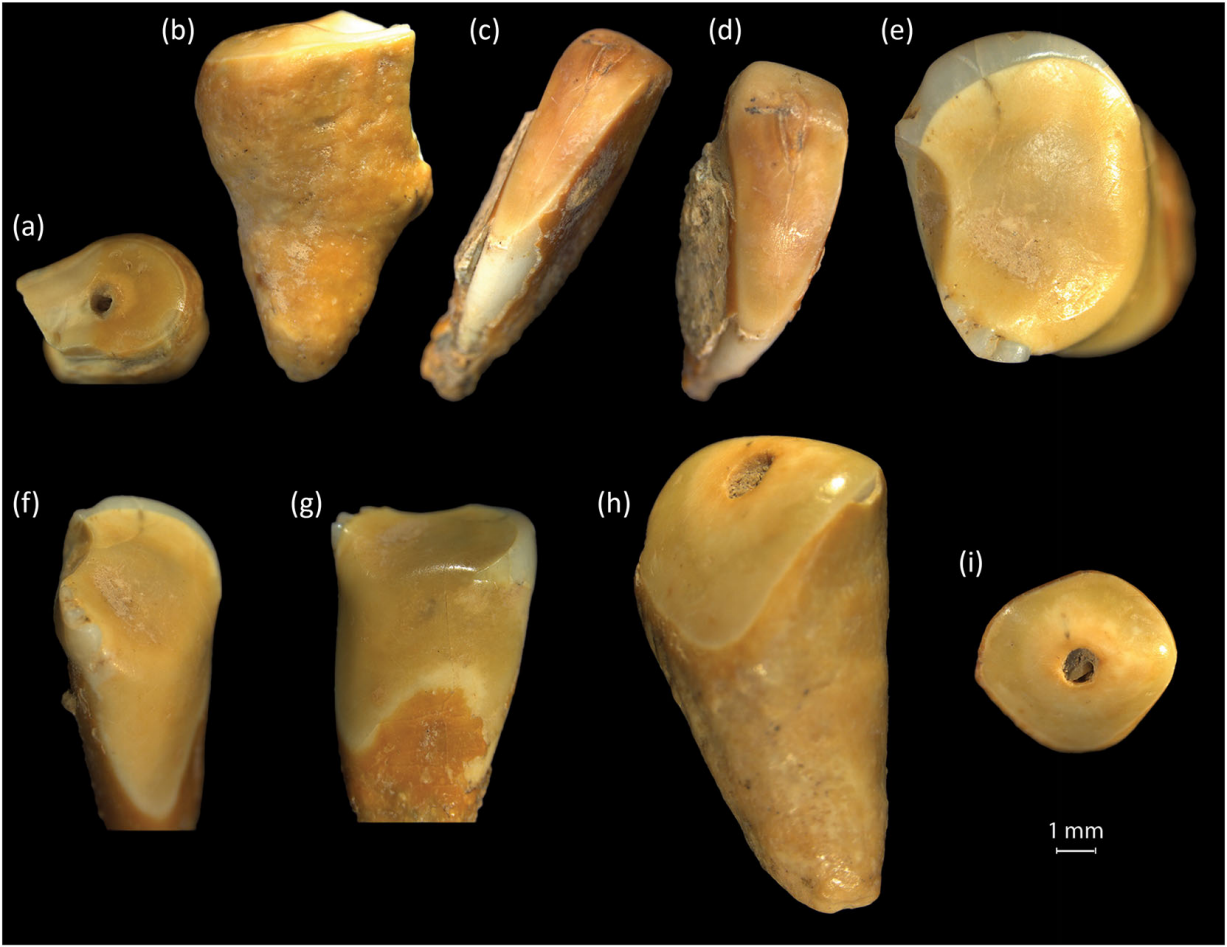
Examples of extreme wear to roots and sides of teeth: (a,b) tooth 902 (SK1970) part of a molar; (c,d) tooth 890 (SK2112) lower incisor with lingual wear; (e–g) tooth 899 (SK2112) lower premolar with wear to three aspects of the root surface; (h,i) tooth 770 (SK1775) an upper first incisor worn to the pulp chamber [Colour figure can be viewed at wileyonlinelibrary.com]

A total of 39 adult teeth (6.6%) from 10 individuals showed extreme levels of wear scoring 7–8 using Molnar (1971) with the roots functioning as tooth surfaces (see Figure 3). Two individuals (SK2286 and SK2373) each had nine extremely worn teeth; both are older probable females. Five isolated teeth, potentially from one individual also had root resorbtion, which may be caused by degeneration, or periodontal trauma (Hillson, 1996, p. 206; Nelson, 2016, p. 222).

### 3.2 Notches and grooves

**FIGURE 4:**
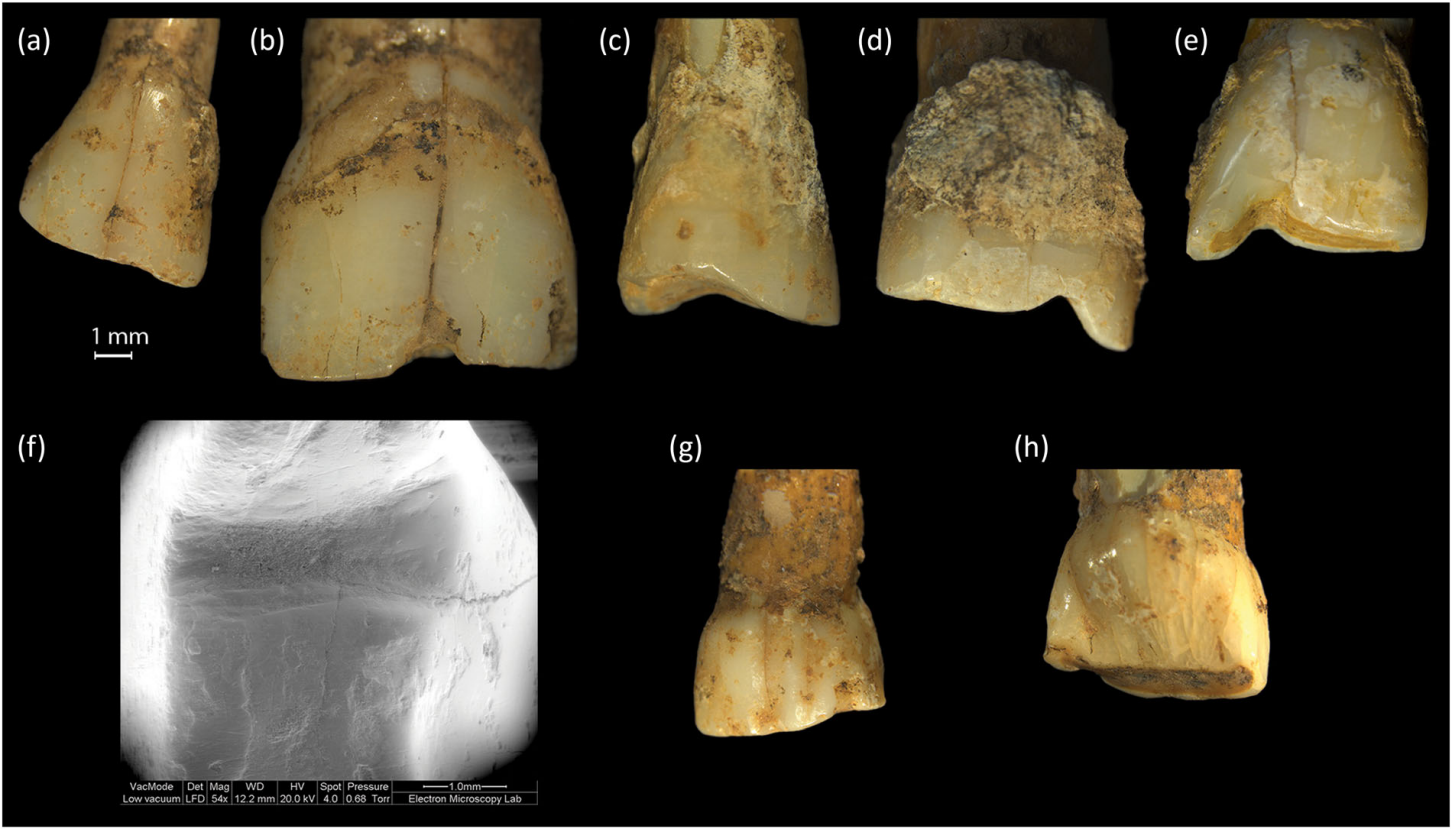
Examples of anterior notching from slightest to the most severe (from right to left) (a) slight notching of tooth 598 (SK1625‐1) an upper right I2; (b) moderate notching of tooth 596 (SK1625‐1) an upper right I1; (c) wide notching of tooth 709 (SK1866) an upper right I2, oblique view; (d–f) most severe notching, tooth 711 (SK1866) upper left I1 (d = labial, e = lingual, f = SEM occlusal view of anterior posterior notch, 54× magnification, 12.2‐mm working distance). (g,h) Tooth 714 (SK1868‐2) a deciduous upper first incisor with notch and chip [Colour figure can be viewed at wileyonlinelibrary.com]

A total of 33 permanent and eight deciduous teeth were notched (Table 2; Figure 4) with notches predominantly affecting the upper incisors and canines in both permanent and deciduous teeth (38 anterior: three posterior). The notched permanent teeth were from nine individuals (eight adults, and one adolescent). One adult (SK1778) had six notched teeth. Of the eight deciduous notched teeth most came from two individuals, SK1631‐2 and SK1868‐8, both aged around 6–8 years.

**FIGURE 5:**
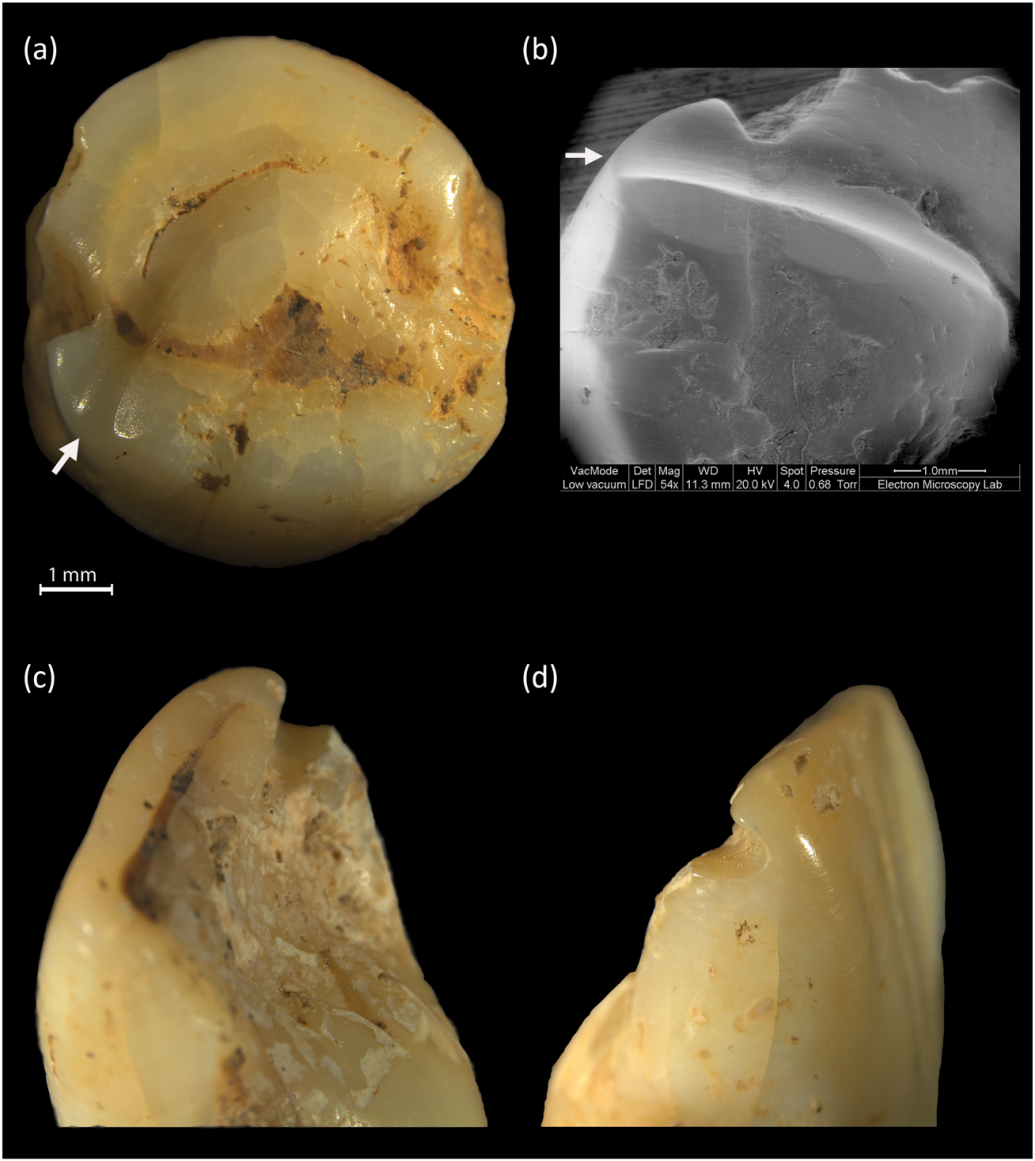
Example of occlusal grooves, (a) tooth 280 (SK1866) lower left premolar 1, occlusal view, (b) SEM image of tooth 280, occlusal surface showing groove and surrounding faceted areas (54× magnification, 11.3‐mm working distance); (c,d) tooth 1175 upper first left incisor, showing proximal and distal views of occlusal groove [Colour figure can be viewed at wileyonlinelibrary.com]

Occlusal grooves were found on 12 permanent teeth (2.6%) including four first premolars, one upper second incisor, two canines, and five upper first incisors (Figure 5). Only one individual, an adult female (SK1788) was affected over four teeth including two first premolars (upper and lower). Two individuals, SK1972‐1 a young adult, and SK1991 an adolescent, had grooves across the anterior occlusal surfaces of both upper first incisors (Erdal, 2008).

### 3.3 Dental chipping, trauma, and abrasion

**FIGURE 6:**
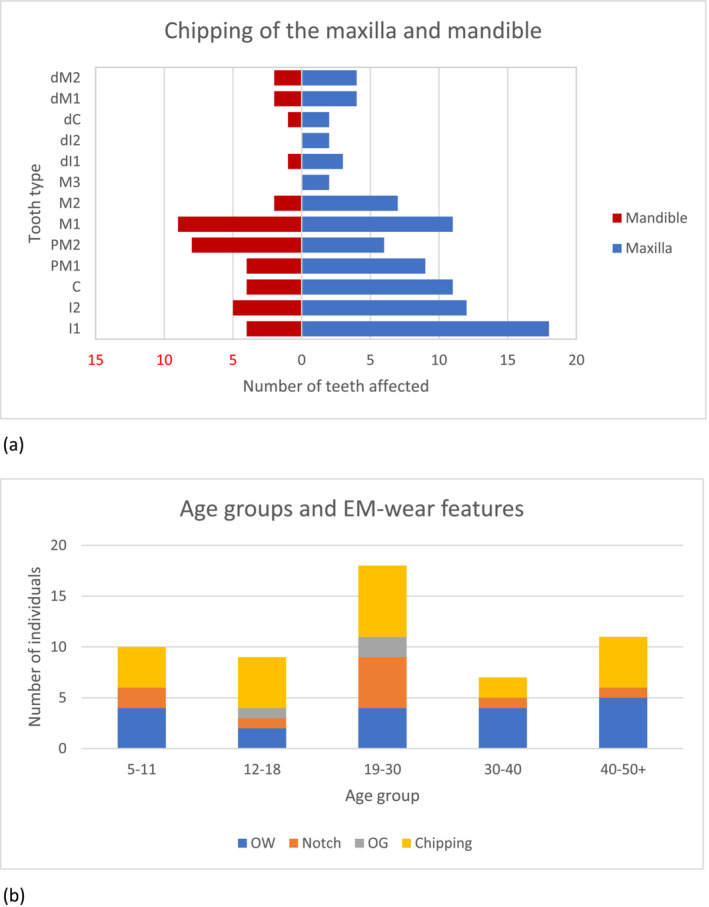
(a) The number and type of maxillary and mandibular teeth affected by chipping. (b) The number of individuals per age group affected by different extra‐masticatory wear features [Colour figure can be viewed at wileyonlinelibrary.com]

**FIGURE 7:**
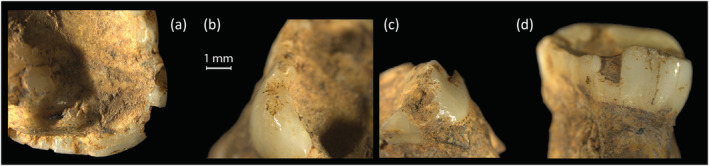
(a–c) Examples of multiple chipping defects to tooth 294 (SK1788) upper left M1 chipped enamel margin of occlusal surface, unworn abrasion, large, chipped defect with adjacent abrasions, (d) tooth 831 (SK1868‐6) deciduous upper M1 with worn chip to lingual aspect [Colour figure can be viewed at wileyonlinelibrary.com]

There are a variety of chipping alterations across all tooth types except third molars; these features range in size from around 1 to 3 mm in area. Chipping affected a total of 136 teeth (see Table S3), of which 59 were left, 73 were right, and four were unsided, potentially showing a slight bias in the side affected. Chipped teeth were seen in both males and females; the ages of the 18 affected individuals range from 5 to 50+ years. In both the permanent and deciduous dentition, upper teeth were more affected by chipping (Table S3; Figure 6a). Chipping is present on teeth with both flat and angled wear. These features are most often fractures of the enamel only, where the incisal or occlusal edge is chipped (Figure 7).

## 4 DISCUSSION

Most of the dental wear features discussed in this assemblage co‐occur in 12 of the total 27 affected individuals. In individuals with multiple features of extra‐masticatory wear, it is much more likely they used their teeth for extra‐masticatory activities. The clearest example is an adult female (SK1778) aged around 20 to 30 years. This individual has the most dental wear features with 24 of 30 teeth affected. This includes six notched teeth, two teeth with occlusal grooves, and 14 teeth with chipping of the enamel and differential wear to the first upper molars.

The early stages of extra‐masticatory dental wear features are difficult to identify (Sperduti et al., 2018), which leads to consideration of how features such as notches and grooves develop, progress, and wear away over time. For example, Figure 4 illustrates variation in notches, with the teeth in Figure 4a,b being from the same individual aged around 20–30 years (SK1625‐1). Comparing Figure 4c with Figure 4d shows how notches can also progress differently in shape.

**FIGURE 8:**
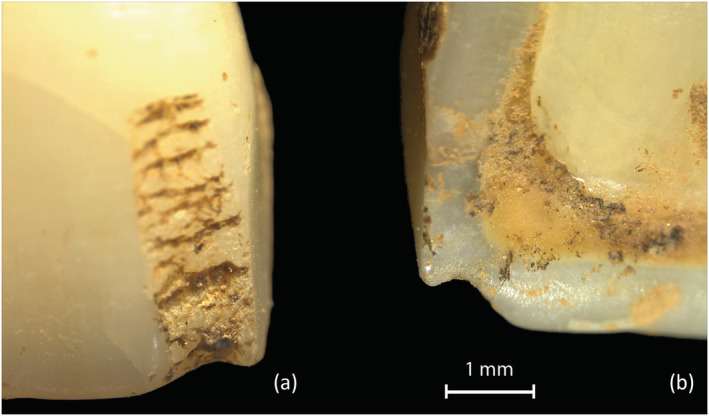
Tooth 556 (SK1781) upper right I1, (a) labial view of abraded chipped area at mesial edge, (b) lingual view of smooth notch to mesial [Colour figure can be viewed at wileyonlinelibrary.com]

In some specimens, it is unclear if notches developed after chipping of the enamel. For example, Figure 8a,b shows a vertical chip with worn edges and an abraded area to the labial enamel associated with a worn notch to the incisal edge of an upper first incisor. Of the total 46 teeth with chipping, 13 of the chip defects are vertical abrasions, some are more worn than others, but all have some rounding to the defect margins. It would require a larger sample to assess the progression of extra‐masticatory wear over time, and these differences may result from the use of different objects or materials of different coarseness or size. This possible progression of features has not been discussed in other studies.

The results of the study show that there is no bias in the age or sex of the affected individuals. Schulz (1977) showed a similar lack of sex bias, whereas Larsen (1985) and Lukacs and Pastor (1988) had male bias in dental wear features.

In terms of specific features and age bias, oblique wear, notches, and chipping affected individuals aged from 5 to 50+ years; this includes deciduous teeth. A source of uncertainty especially in the deciduous teeth is diet; this would not rule out features like chipping but could make the enamel more susceptible to wear.

The number of affected deciduous teeth was unexpected as this has not been observed in previous studies. Of the 20 juveniles in the study, 10 showed features of extra‐masticatory wear. Chipping affects one or multiple teeth in these individuals. For example, a juvenile aged around 6 to 8 years old (SK1631‐2) has 13 affected deciduous teeth, with wear comparable to Molnar's (1971) stages 4 and 5. Of these teeth, three incisors are notched, and four teeth have chipping. The examples of notching and chipping indicate that some children aged from around 5 years upwards were taking part in activities that required them to use their teeth as tools (Figure 7d).

Once all the individuals are split into age groups, these subsamples become too small to be informative; a larger sample is necessary for any further interpretation. However, the most affected age group is adults from around age 19 to 30 (Figure 6b). This is likely to relate to the progression of masticatory wear, with subtler features like grooves and notches being worn away with increased age.

The most frequent of features indicative of tooth use for activities are notches and chipping. Notches indicate the repeated use of the anterior teeth, and there are individuals with notched upper and lower teeth. Both occlusal grooves and notches are thought to be caused by processing fibrous materials that are pulled through the teeth (Willman et al., 2021). This could be for making baskets (Larsen, 1985), reed mats (Minozzi et al., 2003; Molleson, 1994), nets or ropes (Schulz, 1977), working sinew (Waters‐Rist et al., 2010), or wool fibers (Lozano et al., 2021). The presence of pierced net sinkers, reed matting, and beads at Bestansur all support the use of teeth for processing fibers relating to the creation of matting, fishing nets, and threaded bead ornaments (Matthews et al., 2019). Notches can also be caused by the grasping and biting of hard materials, such as bone, wood, or stone tools (Alt & Pichler, 1998), or food items such as seeds (Kaidonis et al., 2012).

Dental chipping can also be caused by activities relating to these items (Molleson & Jones, 1991; Molnar, 2008; Turner & Cadien, 1969), in addition to other causes of trauma (Lovell & Grauer, 2018, p. 347). Generally, dental chipping is thought to affect the molars more in hunter‐gatherers, and the anterior teeth in agriculturalists (Scott & Winn, 2011; Towle et al., 2017). Anterior chipping is more likely to be associated with nonmasticatory activities such as lithic retouching or clamping the teeth while applying force to an object (Hinton, 1981; Willman, 2016, p. 50).

At Bestansur, the greater number of maxillary and anterior teeth affected indicates extra‐masticatory activities as a cause, although some of these activities may have been related to food preparation.

Within the literature, oblique wear planes have been associated with a less abrasive diet and flat wear planes to a more fibrous diet (Fidalgo et al., 2020; Smith, 1984). These differences have been used to differentiate the dentitions of hunter‐gatherers and agriculturalists, with the latter having increased angled wear (Eshed et al., 2006).

Of the 20 individuals from Bestansur with oblique wear, all but three had other indications of extra‐masticatory wear, most frequently notches and chipping. The co‐occurrence of these features within individuals points to either varied extra‐masticatory practices, or perhaps more likely a combination of extra‐masticatory practices alongside dietary alterations to the teeth.

The occlusal wear in the assemblage is varied in direction and extremity. It is possible that the variation in occlusal wear planes may reflect the varied dietary resources cultivated in the area at this time. Archeological evidence from Bestansur indicates use of a broad range of animals, both wild and domesticated (Bendrey et al., 2020). Evidence of plant foods being processed at Bestansur include cereal grains such as glume and free threshing wheats, and wild plant remains including nuts and fruits (Whitlam et al., 2020). Similar dietary resources have been identified at other sites in the region including Ganj Dareh, Abdul Hosein, and Choga Golan (Merrett et al., 2021; Weide et al., 2017).

Ground stone tools from Bestansur used for processing grains would also have caused inclusions in resulting foods. Unfortunately, the effects of specific foods and inclusions from food processing are difficult to isolate archeologically without preservation of these materials. While there has been experimental and ethnological work on food processing tools and resulting inclusions, the mechanical and physiological effects of these materials on human dentition are not clear (Alonso et al., 2020; Valamoti et al., 2020). The effect of ground stone tools could be analysed through dental microwear analysis in future (Hernando et al., 2020). The variation in dental wear patterns seen in this assemblage could fit with a population practicing early agriculture, which is supplemented by hunter‐gatherer practices; further studies are needed to confirm this.

## 5 CONCLUSION

In this early Neolithic community at Bestansur, evidence of extra‐masticatory wear shows that individuals from age 5 upwards took part in activities that required the use of their teeth. These activities may have included the creation of netting for fishing, the weaving of reeds or similar materials, and the processing of fibers for threading items such as beads.

The inclusion of the deciduous dentition in this analysis demonstrates the age at which younger individuals undertook activities in this society. The varied indications of extra‐masticatory wear may reflect the mixed diet and practices of this transitional community.

This study shows the wealth of data that can be obtained when conducting in‐depth analysis of all aspects of dental wear. The development and progression of masticatory and extra‐masticatory wear and its relationship with diet throughout the life course need future consideration.

## CONFLICTS OF INTEREST

The author confirms there are no conflicts of interest.

## Supporting information


**Table S1:** Sex estimation of individuals with dentition (NP = not possible, NA = not applicable)
**Table S2:** Number and percentage of wear planes of permanent and deciduous teeth.
**Table S3:** Number and percentages of chipped teeth from maxillary and mandibular dentitions

## Data Availability

Further data that support the findings in the study are in the supporting information.
