# Supplementary material for: Early evidence of extra‐masticatory dental wear in a Neolithic community at Bestansur, Iraqi Kurdistan
Source: Int J Osteoarchaeol. 2022 Sep 27;32(6):1264–74. doi: 10.1002/oa.3162 (PMC10087735; doi:10.1002/oa.3162)
Supplement: Supplementary file 1 — Table S1: Sex estimation of individuals with dentition (NP = not possible, NA = not applicable) Table S2: Number and percentage of wear planes of permanent and deciduous teeth. Table S3: Number and percentages of chipped teeth from maxillary and mandibular dentitions [file OA-32-1264-s001.docx]

Supplementary information

Running head: Extra-masticatory dental wear at Neolithic Bestansur, Iraqi Kurdistan

Table S1: Sex estimation of individuals with dentition (NP = not possible, NA = not applicable)

| **Sex** | **Number** |
| --- | --- |
| F/F? | 10 |
| M/M? | 10 |
| ? | 1 |
| NP | 2 |
| NA | 15 |
| **Total** | **38** |

Table S2: Number and percentage of wear planes of permanent and deciduous teeth.

|  | **Permanent** | | **Deciduous** | |
| --- | --- | --- | --- | --- |
| **Wear plane** | **N** | **%** | **N** | **%** |
| Unworn | 159 | 35.1 | 47 | 40.8 |
| Buccal-lingual | 80 | 17.6 | 16 | 13.9 |
| Lingual-buccal | 25 | 5.5 | 2 | 1.7 |
| Mesial-distal | 37 | 8.1 | 3 | 2.6 |
| Distal-mesial | 23 | 5 | 10 | 8.7 |
| Flat | 129 | 28.4 | 37 | 32.1 |

Table S3: Number and percentages of chipped teeth from maxillary and mandibular dentitions

|  | **Permanent** | | **Deciduous** | | **Total** | |
| --- | --- | --- | --- | --- | --- | --- |
|  | N | % | N | % | N | % |
| **Maxilla** | 76 | 66 | 15 | 71.4 | 91 | 66.9 |
| **Mandible** | 39 | 33.9 | 6 | 28.5 | 45 | 33 |
